# Prevalence of psychiatric disorders in trauma patients: results from a major trauma unit

**DOI:** 10.1186/cc14557

**Published:** 2015-03-16

**Authors:** M Adlam, A Feehan, V Metaxa

**Affiliations:** 1 King's College Hospital, London, UK

## Introduction

Mental illness has been recognised as a potential risk factor both for intentional and unintentional injury. About 50% of patients presenting with self-inflicting injuries in emergency departments had previous known psychiatric disorder (PD) [1,2], whereas individuals with mental illness were admitted for unintentional injury twice as often as those without [3]. We aimed to assess the prevalence of PD in trauma patients being admitted to a major trauma ICU and compare it with the nontrauma population.

## Methods

We retrospectively reviewed all admissions from January 2010 to December 2013 in a tertiary, mixed ICU that serves a London major trauma centre (MTC) hospital. Data obtained were age, APACHE II score, reason for admission, length of stay (LOS), mortality and a diagnosis of depression, bipolar, self-harm, psychosis, schizophrenia and suicide attempt.

## Results

**Figure 1:**
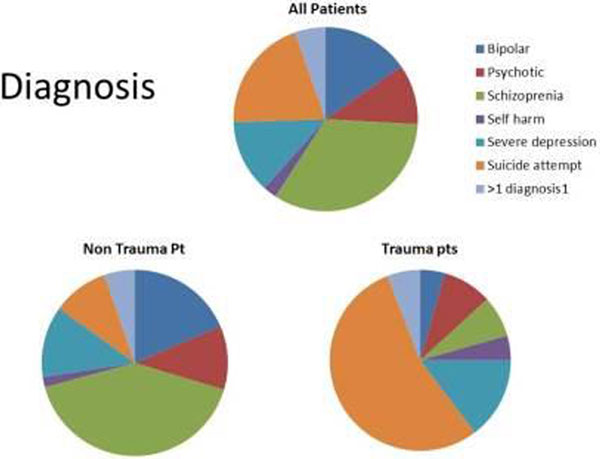


Of 978 trauma patients admitted to the ICU, 68 (7%) had a known PD. Their diagnoses are shown in Figure 1. Median APACHE II score and unadjusted mortality were 13 and 18% respectively in the PD group (15 and 12% in the entire cohort, *P >*0.05). Patients suffering from more than one diagnosis or self-harm alone had increased median LOS (6 vs. 4 days in the entire cohort, *P >*0.05).

## Conclusion

Trauma patients with PD have increased mortality and LOS. MTCs provide a unique opportunity to identify mental illness during hospitalisation through screening and intervention programmes. Integration of mental health services into ICU care should be examined, as it might provide an efficient and cost-effective way of decreasing the risk of reinjury.
